# Reinervação após Denervação Renal – Um Mito?

**DOI:** 10.36660/abc.20210167

**Published:** 2022-07-07

**Authors:** Eric Monteiro, Joana Delgado-Silva, Gonçalo Costa, Lino Gonçalves

**Affiliations:** 1 Centro Universitário e Hospitalar de Coimbra Departamento de Cardiologia Coimbra Portugal Departamento de Cardiologia – Centro Universitário e Hospitalar de Coimbra, Coimbra – Portugal; 2 Universidade de Coimbra Faculdade de Medicina Coimbra Portugal ICBR, Faculdade de Medicina, Universidade de Coimbra, Coimbra – Portugal

**Keywords:** Hipertensão Resistente/terapia, Denervação Renal, Reinervação Renal, Monitoração Ambulatorial da Pressão Arterial/métodos, Diagnóstico por Imagem/métodos

## Introdução

A hipertensão (HTN) é um dos principais fatores de risco que influenciam a carga global de doenças cardiovasculares.^1^ Apesar de medidas como mudanças no estilo de vida e tratamento farmacológico reduzirem a pressão arterial (PA) e as complicações cardiovasculares em todo o mundo, o tratamento da hipertensão permanece sub-ótimo com a PA inadequadamente controlada em muitos pacientes.^2^ No estudo randomizado *ReHOT*, a prevalência de hipertensão resistente foi de 11,7% entre os hipertensos brasileiros, o que está de acordo com a prevalência relatada em outros estudos internacionais.^3,4^ De acordo com as diretrizes atuais da *European Society of HTN*, a HTN resistente é definida quando os valores alvo de PA não são alcançados, apesar da prescrição de terapia tripla, incluindo um diurético na dose máxima tolerada.^5^ Acredita-se que a hiperatividade do sistema nervoso simpático desempenhe um papel importante na HTN resistente. Ao nível renal, a via de saída simpática eferente para os rins leva ao aumento da produção de noradrenalina, vasoconstrição renal e liberação de renina, causando retenção de sódio. Por outro lado, as fibras simpáticas aferentes enviam sinais ao cérebro para estimular a atividade simpática central e contribuem para a HTN neurogênica.^6^ A denervação renal (RDN, *renal denervation*) por cateter surgiu como um dos métodos invasivos mais utilizados para o tratamento da HTN resistente.^7^ Tem como objetivo a ablação dos nervos simpáticos aferentes e eferentes na camada adventícia das artérias renais utilizando energia de radiofrequência. É realizada através da inserção do cateter do dispositivo percutaneamente na artéria femoral, que é então avançado nas artérias renais principais sob orientação fluoroscópica.^6^ De acordo com uma meta-análise, a taxa de complicações do procedimento é baixa, e consiste principalmente em pseudoaneurismas no local de acesso vascular e dissecção da artéria renal.^8^ Entretanto, seu papel na prática clínica é controverso, e há poucas informações sobre as diferentes respostas a esse procedimento.^5^ Relatamos dois casos de HTN resistente idiopática tratados com RDN. Ambos os pacientes apresentaram uma resposta inicial acentuada ao procedimento. No entanto, a PA voltou aos valores basais após 24 e 18 meses de seguimento, respectivamente. Foi realizada uma investigação para detectar causas secundárias de HTN, sem achados que justificassem as alterações da PA. Portanto, foi realizada uma nova RDN, com bons resultados e duração até os dias atuais (6 meses de seguimento para o paciente 1 e mais de três anos de seguimento para o paciente 2). Este é um relato sobre a resposta heterogênea à RDN, o possível papel da reinervação funcional e o potencial desenvolvimento de supersensibilidade à norepinefrina após a RDN. Esses mecanismos podem ser responsáveis por aumentar a PA de volta aos valores basais após uma resposta inicial ideal.

## Relatos de Caso

### Caso 1

Um homem de 49 anos, com histórico de hipertensão, apresentou episódios de tontura e dor torácica associados a picos hipertensivos. O paciente era sedentário, com sobrepeso (altura = 192cm, peso = 98kg, índice de massa corporal – IMC – = 26,6kg/m^2^) e tinha histórico médico de diabetes tipo 2, dislipidemia e gota. Ele fazia uso de cinco medicamentos anti-hipertensivos: amlodipino 5mg/valsartana 80mg duas vezes ao dia, espironolactona 100mg uma vez ao dia, nebivolol 5mg uma vez ao dia e clortalidona 50mg uma vez ao dia. Era tabagista ativo (5 unidades maços/ano) e não tinha histórico de excesso de consumo de álcool ou cafeína. No exame inicial, sua PA no consultório foi de 195/125mmHg, sem disparidade entre os braços. A frequência cardíaca (FC) de repouso foi de 67 batimentos por minuto (bpm) e o restante do exame físico foi normal (bulhas normais, ausência de sopros; pulsos femorais palpáveis bilateralmente; ausência de sopros abdominais). Havia evidência de lesão de órgão mediada por HTN (critérios de hipertrofia ventricular esquerda no ECG – critérios de Sokolov-Lyon 46mm; onda R em aVL 15mm – e hipertrofia concêntrica moderada do ventrículo esquerdo no ecocardiograma – septo interventricular 16mm; parede posterior 12mm; índice de massa do ventrículo esquerdo 134g/m^2^). O paciente havia sido submetido a uma angiografia coronária por TC anterior que não revelou doença coronariana. Foram excluídas as causas secundárias de hipertensão (triagem com perfil bioquímico e hematológico completo, exame de imagem e polissonografia) – ver tabela 1 – e a hipertensão resistente idiopática foi confirmada pela monitorização ambulatorial da pressão arterial (MAPA) – PA média de 24h -159/106 mmHg. A RDN foi proposta e realizada com o cateter multieletrodo Spyral (Medtronic Inc., Santa Rosa, CA, EUA), sem intercorrências. No seguimento de 6 meses, o paciente estava assintomático, havia perdido 6kg ao adotar melhores hábitos de vida (IMC = 24,7kg/m^2^), estava em uso de quatro anti-hipertensivos (nebivolol foi retirado por bradicardia sinusal – FC de repouso = 52bpm) e a PA sistólica e diastólica no MAPA mostraram redução para 15 e 10 mmHg, respectivamente (PA média de 24h: 144/96 mmHg). Entretanto, no seguimento de 24 meses, apesar da manutenção da perda de peso, o paciente apresentava PA média de 24h de 181/120 mmHg no MAPA. Sua FC de repouso era de 70bpm e o nebivolol foi reintroduzido (o paciente voltou a fazer uso de cinco medicamentos hipertensivos).

**Tabela 1:** Triagem para causas secundárias de hipertensão

		Paciente 1	Paciente 2	Valores de referência
Metanefrinas fracionadas no plasma:				
	Metanefrina (pg/mL)	15,2	31,7	<60
	Normetanefrina (pg/mL)	32,6	9,15	<120
Concentração plasmática do hormônio estimulante da tireoide (uUI/mL)	2,3	1,1	0,4-4,0
Atividade da renina plasmática (ng/mL/h)	1,76	1,29	1-4
Concentração plasmática de aldosterona (ng/dL)	32,1	3,42	5-30
Relação aldosterona-renina	18,21	2,65	<25
Concentração de creatinina sérica (mg/dL)	0,99	0,75	Mulheres: 0,55-1,02 Homens: 0,72-1,18
Análise de urina	Negativo para proteínas, eritrócitos e leucócitos	Negativo para proteínas, eritrócitos e leucócitos	NA
Polissonografia (IAH)	3,2	7,6	<5
Angiotomografia Computadorizada	Sem estenose hemodinamicamente significante	Sem estenose hemodinamicamente significante	NA
Concentração sérica de paratormônio (pg/mL)	26	18	9-72
Concentração de cálcio sérico (mg/dL)	9,8	9,3	8,8-10,6
Cortisol salivar 23.00h (ug/dL)	0,087	0,127	<0,15

IAH: índice de apneia e hipopneia; NA: não aplicável.

### Caso 2

Uma mulher de 74 anos apresentou episódios de cefaleia associados a picos hipertensivos e sonolência diurna excessiva. A paciente era sedentária, com sobrepeso (altura = 155cm, peso = 63kg, IMC = 26,2kg/m^2^) e tinha histórico médico de hipertensão e dislipidemia. Fazia uso de quatro medicamentos anti-hipertensivos: nifedipina 60mg pela manhã e 30mg ao jantar, perindopril 5mg duas vezes ao dia, carvedilol 12,5mg duas vezes ao dia e clortalidona 50mg uma vez ao dia. A paciente não tinha histórico de tabagismo, excesso de consumo de álcool ou cafeína. Ao exame físico, a PA no consultório foi de 200/90 mmHg, sem disparidade entre os braços. A FC de repouso era de 58 bpm e o restante do exame físico foi normal (bulhas cardíacas normais, ausência de sopros; pulsos femorais palpáveis bilateralmente; ausência de sopros abdominais). Não havia evidência de lesão de órgão mediada por HTN (septo interventricular 9mm; parede posterior 9mm; índice de massa do ventrículo esquerdo 79g/m^2^). Uma angiotomografia renal prévia revelou placas ateromatosas em ambos os óstios das artérias renais, mas sem estenose hemodinamicamente significante. As causas secundárias de HTN foram avaliadas (Tabela 1), revelando uma leve apneia obstrutiva do sono. No entanto, os valores do MAPA não melhoraram com a pressão positiva contínua nas vias aéreas, apesar da aderência confirmada – PA média de 24h: 158/79 mmHg. A RDN foi proposta e realizada com o cateter multieletrodo Spyral (Medtronic Inc., Santa Rosa, CA, EUA), sem intercorrências. Aos 6 meses de seguimento, a paciente não apresentava sintomas cardiovasculares. Ela apresentava o mesmo IMC e ainda fazia uso de quatro medicamentos anti-hipertensivos, mas o MAPA apresentava PA média de 24h de 110/60 mmHg (redução sistólica e diastólica de 48 e 19 mmHg, respectivamente). Entretanto, aos 18 meses de seguimento, a paciente apresentou novo episódio hipertensivo (PA de 190/85 mmHg). Um novo MAPA foi realizado e revelou PA média de 24h de 146/70 mmHg.

### Investigações e tratamento

**Figura 1:**
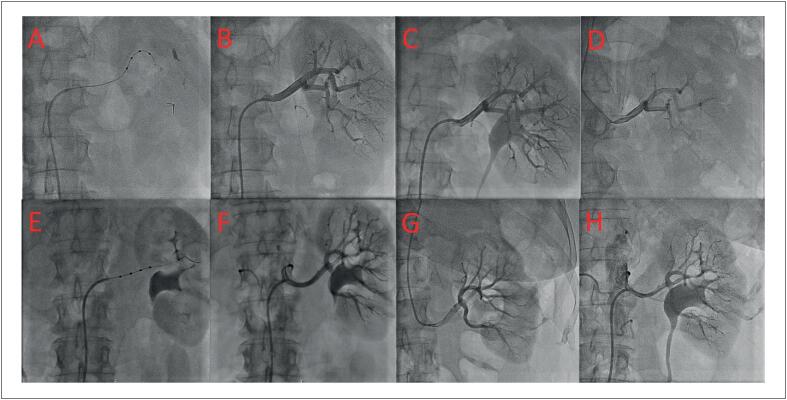
Avaliação das artérias renais. Painéis A-D) caso 1: artéria renal esquerda pré-1ª RDN, imediatamente pós-1ª RDN, aos 6 meses de seguimento após a 1ª RDN e imediatamente após a 2ª RDN, respectivamente; Painéis E-H) caso 2: artéria renal esquerda pré-1ª RDN, imediatamente pós-1ª RDN, aos 6 meses de seguimento após a 1ª RDN e imediatamente pós-2ª RDN, respectivamente. Apenas a artéria renal esquerda de cada paciente é mostrada. A artéria renal contralateral estava em condições semelhantes. RDN: denervação renal.

Os pacientes foram reavaliados para causas secundárias de hipertensão, mas nenhuma causa foi encontrada. Uma nova RDN foi proposta, sendo aceita pelos pacientes. Ambos os procedimentos foram realizados através da artéria femoral, utilizando o cateter multieletrodo Spyral (Medtronic Inc., Santa Rosa, CA, EUA), sem complicações relacionadas ao procedimento (Figura 1).

### Desfecho e seguimento

#### Caso 1

Seis meses após o segundo procedimento, a média da PA de 24h registrada pelo MAPA foi de 159/103mmHg (queda da PA sistólica e diastólica de 22 e 17 mmHg, respectivamente). O paciente estava assintomático com peso estabilizado e não houve recorrência de bradicardia sinusal. A medicação anti-hipertensiva não foi mudada.

**Figura 2:**
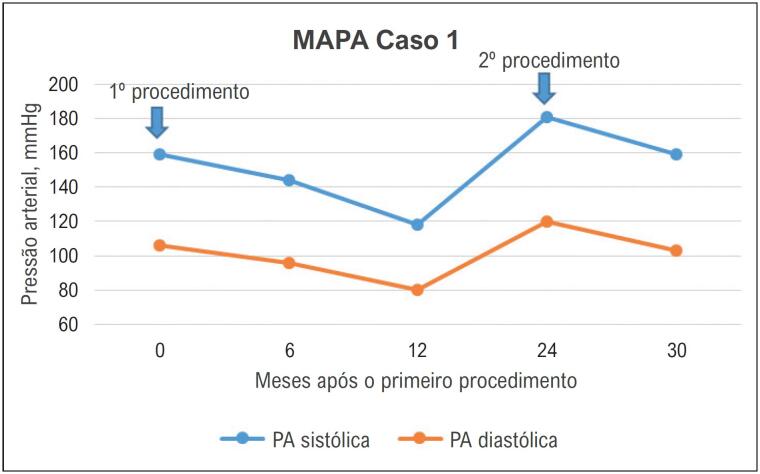
Evolução da pressão arterial do caso 1 registrada pela monitorização ambulatorial da pressão arterial, antes e após a denervação renal. MAPA: monitorização ambulatorial da pressão arterial; PA: pressão arterial.

A resposta da PA antes e depois de ambos os procedimentos de RDN é ilustrada na figura 2.

#### Caso 2

Aos 6 meses de seguimento do segundo procedimento, a média da PA de 24h registrada pela MAPA foi de 127/68mmHg (queda da PA sistólica e diastólica de 19 e 2 mmHg, respectivamente). A PA permaneceu estável no seguimento de 1 ano, 2 anos e 3 anos. Nesse período, a medicação anti-hipertensiva da paciente foi progressivamente reduzida devido aos episódios hipotensores. No geral, o estado geral da paciente melhorou, sem registro de sintomas ou sinais hipertensivos até os dias atuais.

**Figura 3:**
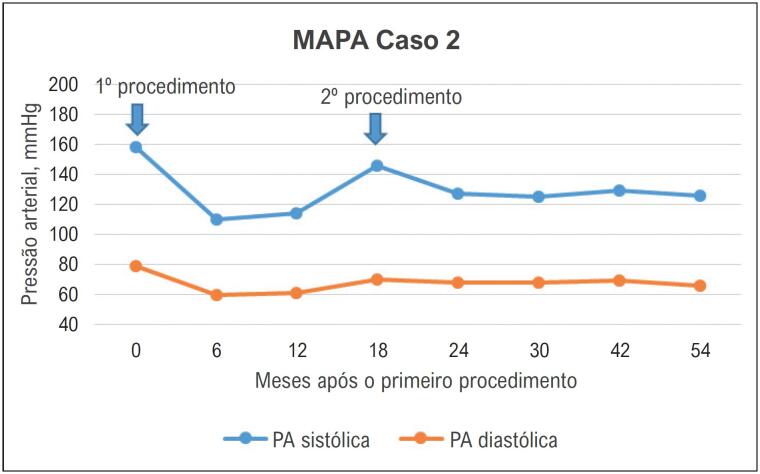
Evolução da pressão arterial do caso 2 registrada pela monitorização ambulatorial da pressão arterial, antes e após a denervação renal. MAPA: monitorização ambulatorial da pressão arterial; PA: pressão arterial.

A resposta da PA antes e depois de ambos os procedimentos de RDN é ilustrada na figura 3.

## Discussão

Acredita-se que as limitações das estratégias farmacológicas disponíveis para controlar a PA em alguns pacientes reflitam a complexidade e a multiplicidade de mecanismos potenciais responsáveis pela gênese e manutenção da PA elevada. Isso levou a um interesse renovado pelas estratégias invasivas.^9,10^ Os nervos simpáticos renais contribuem para o desenvolvimento e perpetuação da HTN, e o fluxo simpático para os rins é ativado em pacientes com HTN essencial.^11^ A ativação crônica do sistema nervoso simpático constitui um mecanismo central na hipertensão resistente e tem sido alvo da RDN percutânea.^10^

Há evidências robustas derivadas de estudos controlados por simulação bem desenhados e rigorosamente conduzidos (SPYRAL HTN-OFF MED, SPYRAL HTN-ON MED e RADIANCE-HTN SOLO) que apoiam a eficácia e segurança da RDN.^12–14^ No entanto, os resultados disponíveis são apenas em curto prazo, ainda faltando informações sobre a eficácia em longo prazo.^15^ Há poucas informações sobre a extensão da reinervação após a RDN baseada em cateter em humanos, mas estudos em modelos animais mostram evidências de reinervação funcional e anatômica do nervo renal, juntamente com a supersensibilidade à norepinefrina relacionada à denervação. Um estudo realizado em ovelhas avaliou a eficácia da denervação do nervo renal com o cateter Symplicity Flex e a reinervação funcional e anatômica aos 5,5 e 11 meses pós-denervação. Verificou-se que o procedimento efetivamente resultou em denervação dos nervos renais aferentes e eferentes, mas aos 11 meses pós-RDN havia evidências funcionais e anatômicas de reinervação do nervo renal aferente e eferente.^16^ Da mesma forma, um estudo realizado em ratos indica que após a RDN, a reinervação funcional da vasculatura renal começa a ocorrer entre 14 e 24 dias após o procedimento, e esse retorno completo da função pode ocorrer em 8 semanas. O estudo também sugeriu que a resposta à estimulação do nervo renal durante a reinervação pode ocorrer devido a uma combinação de regeneração das fibras nervosas e supersensibilidade à norepinefrina relacionada à denervação.^17^ Embora os resultados finais de três anos do estudo *Symplicity HTN-1*^18^ sugiram que nenhuma reinervação ou quaisquer mecanismos contrarreguladores se desenvolvam ao longo do tempo que possam diminuir a eficácia do procedimento, os dois casos que relatamos, juntamente com as evidências disponíveis em modelos animais, parecem indicar que isso pode não ser universalmente verdadeiro. O fato de que ambos os casos descritos aqui mostraram uma resposta acentuada da PA à primeira RDN, seguida de novo aumento da PA aos valores basais no seguimento, poderia indicar que a reinervação desempenha um papel clinicamente significativo na eficácia de longo prazo do procedimento. Além disso, ambos os pacientes responderam a um procedimento de repetição, fato que parece validar ainda mais essa hipótese.

Considerando esses aspectos conjuntamente, o objetivo deste artigo é levantar questões relacionadas à possibilidade de reinervação e desenvolvimento de supersensibilidade à norepinefrina após a RDN. É crucial saber se a reinervação ocorre, se ela influencia os resultados da intervenção em longo prazo e em qual subgrupo de pacientes esse fenômeno é mais provável de ocorrer.

## Conclusões

Muitos pacientes não são capazes de atingir os valores-alvo de pressão arterial apesar das mudanças no estilo de vida e do tratamento farmacológico.

A denervação renal por cateter apresenta-se como uma alternativa segura e eficaz para este subconjunto de pacientes com hipertensão resistente.

Os dois casos relatados aqui, juntamente com as evidências disponíveis em modelos animais, podem indicar que a reinervação pode desempenhar um papel significativo na eficácia do procedimento em longo prazo.

Portanto, é crucial saber se a reinervação ocorre, se ela influencia os resultados da intervenção em longo prazo e em qual subgrupo de pacientes esse fenômeno é mais provável de ocorrer.
