# Supplementary material for: Reinervação após Denervação Renal – Um Mito?
Source: Arq Bras Cardiol. 2022 Jul 7;119(1):128–32. [Article in Portuguese] doi: 10.36660/abc.20210167 (PMC9352135; doi:10.36660/abc.20210167)
Supplement: Translation

# Translation: Reinnervation after Renal Denervation – A Myth?

**Authors:** Eric Monteiro, Joana Delgado-Silva, Gonçalo Costa, Lino Gonçalves

**Affiliations:** 1 Coimbra Portugal Departamento de Cardiologia – Centro Universitário e Hospitalar de Coimbra, Coimbra – Portugal; 2 Coimbra Portugal ICBR, Faculdade de Medicina, Universidade de Coimbra, Coimbra – Portugal

**Keywords:** Resistant Hypertension/therapy, Renal Denervation, Renal - Re-Innervation, Blood Pressure, Monitoring Ambulatory/methods/ Diagnostic, Imaging/methods

## Introduction

Hypertension (HTN) is a leading risk factor influencing the global burden of cardiovascular disease.^1^ In spite of the fact that measures such as lifestyle changes and pharmacological treatment reduce blood pressure (BP) and cardiovascular complications, worldwide, the treatment of HTN remains suboptimal with inadequately controlled BP in many patients.^2^ In the ReHOT Randomized Study, the prevalence of resistant HTN was 11.7% among Brazilian hypertensive patients, which is in agreement with the prevalence reported in other international studies.^3,4^ According to the current guidelines of the European Society of HTN, resistant HTN is defined when target BP values are not reached, despite prescription of triple therapy, including a diuretic at a maximum tolerated dose.^5^ Sympathetic nervous system hyperactivity is thought to play a major role in resistant HTN. At the kidney level, the efferent sympathetic outflow to the kidneys leads to increased noradrenaline production, renal vasoconstriction and renin release, causing sodium retention. On the other side, afferent sympathetic fibers send signals to the brain to stimulate central sympathetic activity and contribute to neurogenic HTN.^6^ Catheter-based renal denervation (RDN) has emerged as one of the most frequently used invasive methods for the treatment of resistant HTN.^7^ It aims to ablate the afferent and efferent sympathetic nerves in the adventitia of the renal arteries using radiofrequency energy. It is performed through the insertion of the device catheter percutaneously into the femoral artery, which is then advanced into the main renal arteries under fluoroscopic guidance.^6^ According to a meta-analysis, the rate of procedural complications is low and consists mainly in pseudoaneurysms at the vascular access site and renal artery dissection.^8^ Nevertheless, its role in clinical practice is controversial and there is scarce information about the different responses to this procedure.^5^ We report two cases of idiopathic resistant HTN treated with RDN. Both patients had a profound initial response to the procedure. Nevertheless, their BP was back to baseline values at the 24 and 18-month follow-up, respectively. An investigation to detect secondary causes of HTN was performed with no findings that justified the BP changes. Therefore, a new RDN was performed, with good results, lasting until the present day (6-month follow-up for patient 1 and more than 3-year follow-up for patient 2). This is a report about the heterogeneous response to RDN, the possible role of functional re-innervation and the potential development of supersensitivity to norepinephrine after RDN. These mechanisms could be responsible for increasing the BP back to baseline values after an optimal initial response.

## Case reports

### Case 1

A 49-year-old man with a history of HTN, presented with episodes of dizziness and chest pain associated with hypertensive peaks. The patient was sedentary, overweight (height = 192cm, weight = 98kg, body mass index – BMI – = 26.6kg/m^2^) and had a medical history of type 2 diabetes, dyslipidemia and gout. He was on five antihypertensive drugs: amlodipine 5mg/valsartan 80mg bid, spironolactone 100mg od, nebivolol 5mg od and chlortalidone 50mg od. He was an active smoker (5 pack-units-year) and had no history of alcohol or caffeine excess. On initial examination his office BP was 195/125mmHg, with no inter-arm disparity. His resting heart rate (HR) was 67 beats per minute (bpm) and the remaining physical examination was normal (normal cardiac sounds, absence of murmurs; palpable femoral pulses bilaterally; absence of abdominal bruits). There was evidence of HTN-mediated organ damage – HMOD – (left ventricular hypertrophy criteria on ECG – Sokolov-Lyon criteria 46mm; R wave in aVL 15mm – and moderate concentric hypertrophy of the left ventricle on echocardiography – interventricular septum, 16mm; posterior wall, 12mm; left ventricular mass index 134g/m^2^). The patient had undergone a previous CT coronary angiogram that revealed no coronary disease. Secondary causes of HTN were excluded (screening with full biochemistry and hematology profile, imaging assessment and polysomnography) – see table 1 – and idiopathic resistant HTN was confirmed by ambulatory blood pressure monitoring (ABPM) – 24h average BP 159/106 mmHg. RDN was proposed and performed with the multielectrode Spyral catheter (Medtronic Inc., Santa Rosa, CA, USA), without complications. At the 6-month follow-up, the patient was asymptomatic, had lost 6kg by adopting better lifestyle habits (BMI = 24.7kg/m^2^), was on four antihypertensive drugs (nebivolol was withdrawn due to sinus bradycardia – resting HR = 52bpm) and systolic and diastolic BP in ABPM had dropped to 15 and 10 mmHg, respectively (24h average BP 144/96 mmHg). Nevertheless, at the 24-month follow-up, despite maintenance of weight loss, the patient had a 24h average BP of 181/120 mmHg in ABPM. His resting HR was 70bpm and nebivolol was reintroduced (the patient was back on five hypertensive drugs).

**Table 1:** Screening for secondary hypertension causes

		Patient 1	Patient 2	Reference Values
Plasma fractionated metanephrines:				
	Metanephrine (pg/mL)	15.2	31.7	<60
	Normetanephrine (pg/mL)	32.6	9.15	<120
Thyroid-stimulating hormone plasma concentration (uUI/mL)	2.3	1.1	0.4-4.0
Plasma renin activity (ng/mL/h)	1.76	1.29	1-4
Plasma aldosterone concentration (ng/dL)	32.1	3.42	5-30
Aldosterone-renin-ratio	18.21	2.65	<25
Serum creatinine concentration (mg/dL)	0.99	0.75	Females: 0.55-1.02 Males: 0.72-1.18
Urine analysis	Negative for protein, erythrocytes and leucocytes	Negative for protein, erythrocytes and leucocytes	NA
Polysomnography (AHI)	3.2	7.6	<5
Computed tomography angiography	No hemodynamically significant stenosis	No hemodynamically significant stenosis	NA
Serum parathyroid hormone concentartion (pg/mL)	26	18	9-72
Serum calcium concentration (mg/dL)	9.8	9.3	8.8-10.6
Salivary cortisol 23.00h (ug/dL)	0.087	0.127	<0.15

AHI: apnea hypopnea index; NA: not applicable.

### Case 2

A 74-year-old woman presented with episodes of headache associated with hypertensive peaks and excessive daytime sleepiness. The patient was sedentary, overweight (height = 155cm, weight = 63kg, BMI = 26.2kg/m^2^) and had a medical history of HTN and dyslipidemia. She was medicated with four antihypertensive drugs: nifedipine 60mg in the morning and 30mg at dinner, perindopril 5mg bid, carvedilol 12.5mg bid and chlortalidone 50mg od. The patient had no history of smoking, alcohol or caffeine excess. On physical examination, her office BP was 200/90 mmHg, with no inter-arm disparity. Her resting HR was 58 bpm and the remaining physical examination was normal (normal cardiac sounds, absence of murmurs; palpable femoral pulses bilaterally; absence of abdominal bruits). There was no evidence of hypertension-mediated organ damage (HMOD): interventricular septum, 9mm; posterior wall, 9mm; left ventricular mass index, 79g/m^2^. A previous CT renal angiogram revealed atheromatous plaques in the ostium of both renal arteries, but without hemodynamically significant stenosis. Secondary causes of HTN were assessed (Table 1), revealing mild obstructive sleep apnea. Nevertheless, the ABPM values did not improve with continuous positive airway pressure, despite confirmed compliance – 24h average BP 158/79 mmHg. RDN was proposed and performed with the multielectrode Spyral catheter (Medtronic Inc., Santa Rosa, CA, USA), without complications. At the 6-month follow-up, the patient had no cardiovascular symptoms. She had the same BMI and was still on four antihypertensive drugs, but the ABPM showed a 24h average BP of 110/60 mmHg (systolic and diastolic reduction of 48 and 19 mmHg, respectively). Nevertheless, at the 18-month follow-up, the patient had a new hypertensive episode (BP of 190/85 mmHg). A new ABPM was performed and revealed a 24h average BP of 146/70 mmHg.

### Investigations and treatment

The patients were reassessed for secondary causes of HTN, but none was found. A new RDN was proposed, which they accepted. Both procedures were performed through the femoral artery, using the multielectrode Spyral catheter (Medtronic Inc., Santa Rosa, CA, USA), without procedural-related complications (Figure 1).

**Figure 1:** Assessment of the renal arteries. Panels A-D) case 1: left renal artery pre-1^st^ RDN, immediately post-1^st^ RDN, at the 6-month follow-up after the 1^st^ RDN and immediately post-2^nd^ RDN, respectively; Panels E-H) case 2: left renal artery pre-1^st^ RDN, immediately post-1^st^ RDN, at the 6-month follow-up after the 1^st^ RDN and immediately post-2^nd^ RDN, respectively. Only the left renal artery of each patient is shown. The contralateral renal artery was in similar conditions. RDN: renal denervation.

### Outcome and follow-up

#### Case 1

Six months after the second procedure, the average 24h BP registered by ABPM was 159/103mmHg (systolic and diastolic BP drop of 22 and 17 mmHg, respectively). The patient was asymptomatic with a stabilized weight and there was no recurrence of sinus bradycardia. Antihypertensive medication remained unchanged.

BP response before and after both RDN procedures is illustrated in figure 2.

**Figure 2:** Case 1 blood pressure evolution recorded by ambulatory blood pressure monitoring, before and after both renal denervation procedures. ABPM: ambulatory blood pressure monitoring; BP: blood pressure.

#### Case 2

At the 6-month follow-up of the second procedure, the average 24h BP registered by ABPM was 127/68mmHg (systolic and diastolic BP drop of 19 and 2 mmHg, respectively). The BP remained stable at the 1-year, 2-year and 3-year follow-up. During this period the patient’s antihypertensive medication was progressively reduced due to hypotensive episodes. Overall, the patient general condition has improved, with no record of hypertensive symptoms or signs up to the present day.

BP response before and after both RDN procedures is illustrated in figure 3.

**Figure 3:** Case 2 blood pressure evolution recorded by ambulatory blood pressure monitoring, before and after both renal denervation procedures. ABPM: ambulatory blood pressure monitoring; BP: blood pressure.

## Discussion

The limitations of available pharmacological strategies to control BP in some patients is thought to reflect the complexity and multitude of potential mechanisms responsible for the genesis and maintenance of elevated BP. This led to a renewed interest in invasive strategies.^9,10^ Renal sympathetic nerves contribute to the development and perpetuation of HTN, and the sympathetic outflow to the kidneys is activated in patients with essential HTN.^11^ The chronic activation of the sympathetic nervous system constitutes a central mechanism in resistant HTN and has been a target of percutaneous RDN.^10^

There is robust evidence derived from well-designed and rigorously conducted sham-controlled studies (SPYRAL HTN-OFF MED, SPYRAL HTN-ON MED, and RADIANCE-HTN SOLO) supporting the efficacy and safety of RDN.^12–14^ Nevertheless, the available results are short-term only, and long-term efficacy information is still lacking.^15^ There is little information regarding the extent of re-innervation following catheter-based RDN in humans, but studies in animal models show evidence of functional and anatomical renal nerve re-innervation, along with denervation-related supersensitivity to norepinephrine. A study conducted in sheep assessed the effectiveness of renal nerve denervation with the Symplicity Flex™ catheter and the functional and anatomical re-innervation at 5.5 and 11-months post-denervation. It was found that the procedure effectively denervated the afferent and efferent renal nerves, but by 11 months post-RDN, there was functional and anatomical evidence of afferent and efferent renal nerve re-innervation.^16^ Similarly, a study conducted in rats indicates that following RDN, functional re-innervation of the renal vasculature begins to occur between 14 and 24-days after the procedure, and that complete return of function may occur by 8 weeks. The study also suggested that the response to renal nerve stimulation during re-innervation could be due to a combination of regeneration of the nerve fibers and denervation-related supersensivity to norepinephrine.^17^ Although the final 3-year results of the Symplicity HTN-1 study^18^ suggest that no re-innervation or any counter-regulatory mechanisms develop over time that could lessen the efficacy of the procedure, the two present cases, along with the evidence available on animal models, seem to indicate that this may not be universally true. The fact that both cases described herein showed marked BP response to the first RDN, followed by re-elevation of the BP to baseline values at follow up, could indicate that re-innervation plays a clinically significant role in the long-term efficacy of the procedure. Additionally, both patients responded to a repeat procedure, a fact that seems to further validate this hypothesis.

Taking these aspects together, the aim of this paper is to raise concerns about the possibility of re-innervation and the development of supersensitivity to norepinephrine after RDN. It is crucial to know whether re-innervation occurs, if it influences the long- term results of the intervention and in which subset of patients this phenomenon is more likely to occur.

## Conclusions

Many patients are not able to reach target blood pressure values despite lifestyle changes and pharmacological treatment.

Catheter-based renal denervation is a safe and effective alternative for this subset of patients with resistant hypertension.

The two cases reported herein, along with the evidence available on animal models, could indicate that re-innervation may play a significant role in the long term efficacy of the procedure.

It is therefore crucial to know whether re-innervation occurs, if it influences the long- term results of the intervention and in which subset of patients this phenomenon is more likely to occur.
